# Oral health and atherosclerotic cardiovascular disease: A review

**DOI:** 10.1016/j.ajpc.2021.100179

**Published:** 2021-04-05

**Authors:** Eugenia Gianos, Elizabeth A. Jackson, Astha Tejpal, Karen Aspry, James O'Keefe, Monica Aggarwal, Ankur Jain, Dipti Itchhaporia, Kim Williams, Travis Batts, Kathleen E. Allen, Clark Yarber, Robert J. Ostfeld, Michael Miller, Koushik Reddy, Andrew M. Freeman, Kenneth E. Fleisher

**Affiliations:** a Division of Cardiology, Lenox Hill Hospital, Northwell Health, New York, NY, United States; b Division of Cardiovascular Disease, Department of Internal Medicine, University of Alabama at Birmingham, Birmingham, AL, United States; c Lifespan Cardiovascular Institute, and Division of Cardiology, Brown University, Alpert Medical School, Providence, RI, United States; d Saint Luke's Mid America Heart Institute and University of Missouri-Kansas City School of Medicine, Kansas City, MI, United States; e Division of Cardiology, University of Florida, Gainesville, FL, United States; f Jeffrey M. Carlton Heart & Vascular Institute, Hoag Memorial Hospital, Newport Beach, CA, United States; g Department of Medicine, Division of Cardiology, Rush University Medical Center, Chicago, IL, United States; h Division of Cardiology, Department of Medicine, Wilford Hall Ambulatory Surgical Center, San Antonio, TX, United States; i Geisel School of Medicine at Dartmouth, Hanover, NY, United States; j Department of Internal Medicine, Montefiore Health System, Bronx, NY, United States; k Division of Cardiology, Department of Medicine, Montefiore Health System, Bronx, NY, United States; l Department of Cardiovascular Medicine, Epidemiology & Public Health, University of Maryland School of Medicine, Baltimore, MD, United States; m Division of Cardiology, James A. Haley VA Medical Center, University of South Florida, Tampa, FL, United States; n Division of Cardiology, Department of Medicine, National Jewish Health, Denver, CO, United States; o Department of Oral and Maxillofacial Surgery, NYU College of Dentistry, New York, NY, United States

**Keywords:** Periodontal disease, Oral health, Cardiovascular prevention, Cardiovascular Disease

## Abstract

Periodontal disease (PD) is common in the US and globally. Evidence suggests that poor oral health is associated with atherosclerotic cardiovascular disease (ASCVD); however, this relationship has not been a major focus in clinical cardiology. This manuscript will review the growing evidence linking PD to ASCVD, including pathophysiologic mechanisms and coexistent risk factors. Public health considerations with a focus on disparities, social determinants, preventive strategies, and a call to action to reduce the burden of coincident ASCVD and PD are also reviewed.

## Highlights

• Mechanistic studies illustrate the effects of PD on systemic inflammation, platelet and endothelial function, and lipoproteins.
• Trials of PD treatment have not shown reductions in cardiovascular outcomes therefore a definite causal association is lacking.
• The morbidity and impact on quality of life, mutual risk factors and systemic inflammation warrant preventive efforts.
• Improved screening, better collaboration, and targeted health policies could greatly improve prevention of PD and its sequelae.

## Graphical abstract

**Figure:**
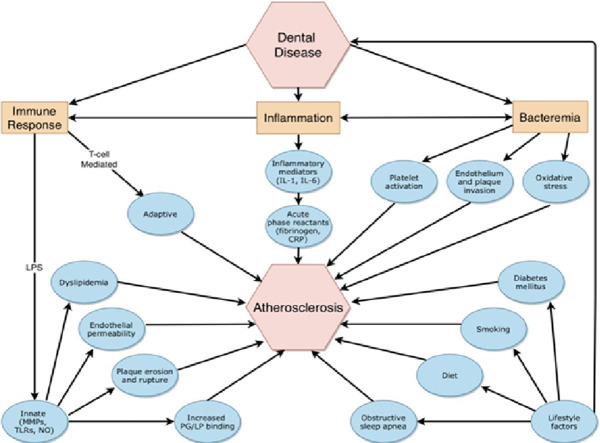

## 1 Introduction

**Fig. 1:**
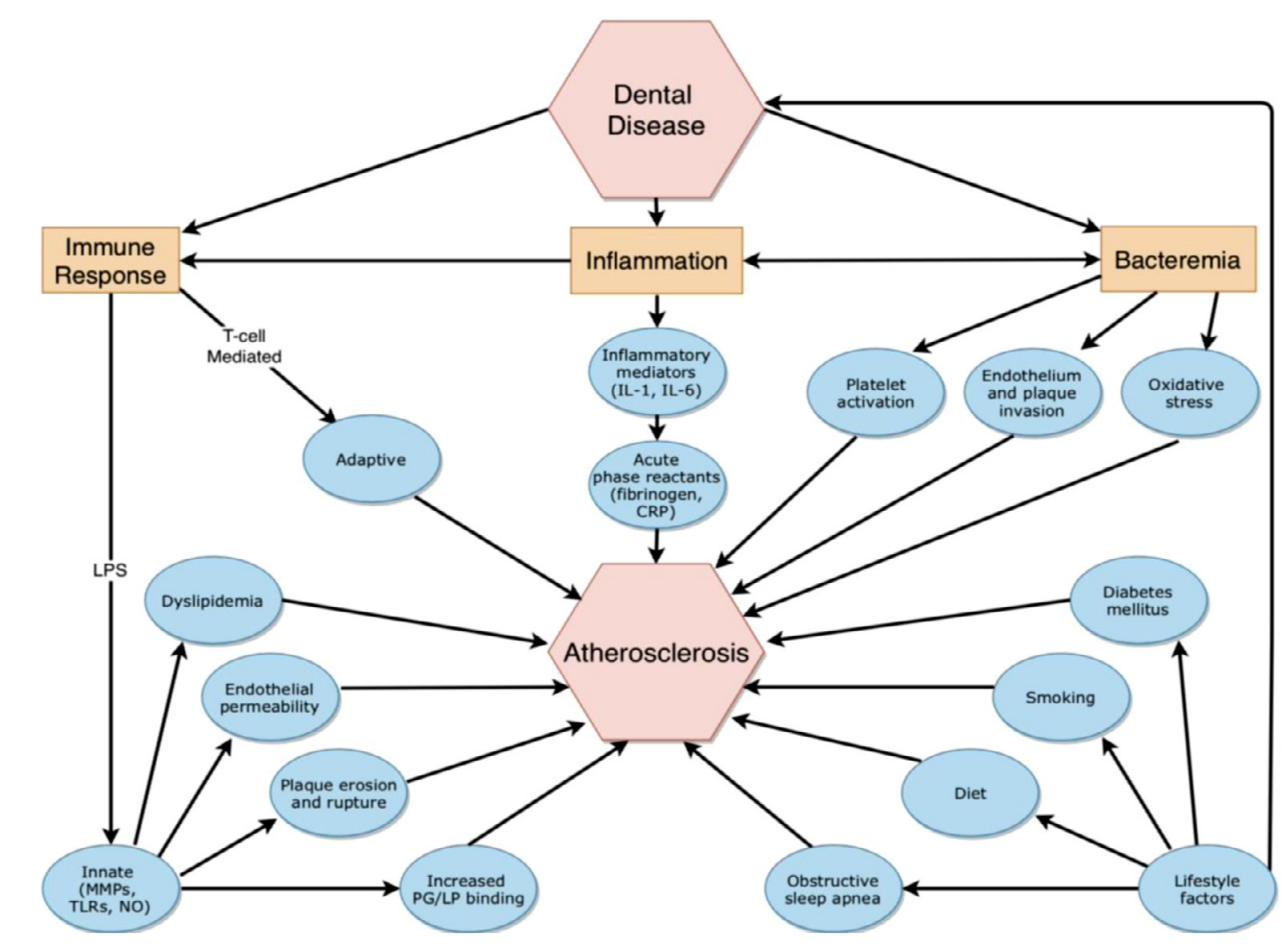
**(central illustration):** Proposed mechanisms linking dental disease and atherosclerosis. IL: Interleukin; CRP: C-reactive protein; MMP: Matrix metalloproteinase; TLR: Toll-like receptor; NO: Nitric oxide; PG: Proteoglycan; LP: Lipoprotein.

Atherosclerotic cardiovascular disease (ASCVD) is a leading cause of disability and death in the U.S. and globally [1]. It is well established that modification of established ASCVD risk factors reduces morbidity and mortality from ASCVD; however, other prevention strategies may also contribute to cardiovascular risk improvements. Periodontal disease (PD) is one of the most prevalent chronic infections, affecting 46% of US adults [2]. In the last 30 years, evidence has accumulated that links PD to ASCVD via inflammatory and immune-mediated mechanisms (Fig. 1). The objective of this narrative review is to summarize the biologic mechanisms and epidemiologic evidence linking poor oral health to ASCVD risk and the role of health inequality.

## 2 Definition and prevalence of periodontal disease

More than 700 oral microbes populate dental surfaces, and dental plaque contains a biofilm that adapts to environmental flora changes [3]. Gingivitis develops when this plaque extends to the surrounding gingiva, triggering an immune response and inflammation. Gingivitis may progress to PD when bacteria and inflammation migrate apically along the root surface and penetrate the tooth's supporting structures, including the surrounding bone. PD can lead to irreversible destruction of connective tissue fibers attached to the tooth, causing bone resorption and tooth loss. PD affects 47% of adults aged 30 years and older and increases with age, occurring in 70% of adults 65 years and older. PD is more common in men than women (56.4% vs 38.4%) and in non-Hispanic blacks and Mexican-Americans. Risk factors for PD include smoking, diabetes, poor oral hygiene, stress, heredity, crooked teeth, compromised immune function, defective fillings, mediations that cause dry mouth, bridges that no longer fit properly and female hormonal changes such as pregnancy or use of oral contraceptives [4,5].

Dysbiosis, defined as an imbalance in oral flora, may trigger bacteremia and facilitate systemic dissemination of oral bacteria [3]. Thus, PD can trigger bacteremia resulting in profound local and systemic inflammatory and immune-mediated responses.^6^  Risk factors for PD, including diabetes, obesity and smoking, are also risk factors for ASCVD thereby confounding the relationship between PD and ASCVD [7].

## 3 Biological mechanisms linking pd to ASCVD

### 3.1 Bacterial translocation

Mechanistic studies have provided evidence for dental infection as a potential mediator of ASCVD. Whether this process is initiated and advanced through direct translocation of bacteria or bacterial products or the downstream inflammatory and immune-mediated mechanisms remains unclear [6]. Bacteremia, of variable degrees, has been confirmed by multiple studies immediately following dental extraction and tooth brushing, which correlates with the systemic markers of inflammation [8]. Periodontal bacteria have also been detected in atheromatous aortic plaques,[9] and in thrombi from patients with acute myocardial infarction (MI),[10] suggesting direct effects of oral microbes at these distant sites. Periodontal bacteria, including *Aggregatibacter actinomycetemcomitans* and *Porphyromonas gingivalis* (*P. gingivalis),* have also been found to penetrate human vascular endothelium and cause endothelial dysfunction via lipopolysaccharide (LPS)-mediated effects [11]. LPS on the outer capsule of periodontal gram-negative bacteria, particularly *P. gingivalis,* participates in numerous other proatherogenic processes discussed below [11].

### 3.2 Inflammatory mechanisms

Inflammatory cells, including neutrophils, monocytes, and macrophages participate in all phases of atherosclerosis [12]. Similar inflammatory pathways have been shown to exist in PD. Pussinen et al. observed the inflammatory process in both atherosclerosis and periodontitis involve local macrophage activation and low-density lipoproteins (LDL)-mediated foam cell formation in the arterial intima and periodontium, respectively [13]. Pro-inflammatory cytokines and endothelial cell adhesion molecules that have been associated with PD in both human and animal studies include C-reactive protein (CRP), tumor necrosis factor-alpha (TNF-α), interleukins (IL), including IL-1β, IL-6, IL-8, and IL-17, matrix metalloproteinases (MMP), vascular cell adhesion molecule-1 (VCAM-1), intercellular adhesion molecule-1 (ICAM-1)*,* E-selectin and P-selectin, most of which have been strongly implicated in the initiation and progression of ASCVD [14], [15], [16]. Studies suggest that the collagenolytic activity found at some chronic inflammation sites, such as periodontitis, derives from MMP-8 which has also been implicated in atherosclerotic plaque destabilization [17]. The PAROKRANK (Periodontitis and its Relation to Coronary Artery Disease) study, a Swedish case-control study of 805 subjects with a first MI and 805 matched controls, reported a positive association between PD and the first presentation of an MI, with a significant correlation in patients with severe periodontitis [18]. It also showed that MMPs are increased in the saliva of patients with gingivitis, but could not demonstrate an association between saliva levels of MMPs and MI incidence. A study by Kodovazenitis et al. evaluated the periodontal status and CRP levels in 87 patients with acute MI and determined that serum levels of CRP were higher in post-acute MI patients with PD versus without PD. This suggests a role for PD-mediated inflammation after controlling for confounding from shared risk factors, including smoking and diabetes [19]. Overall, common inflammatory processes seem to underlie the development of both PD and ASCVD but their specific involvement linking these two disease entities will need to be further examined in future studies.

### 3.3 Immune activation

Similarly, evidence supports the role of PD in triggering immune responses, including macrophage, T-cell and B cell activation [6,20]. Such immune responses are also linked to the development and progression of atherosclerosis [6]. *P. gingivalis* is a potent activator of macrophages and innate immune responses[11] producing Heat Shock Protein, a trigger molecule that links PD and ASCVD [15]. In animal studies, *P. gingivalis* tolerizes resident and infiltrating leukocytes and effectively mutes immune response against it, exacerbating PD [21].

### 3.4 Platelet aggregation and thrombosis

Oral pathogens have also been directly linked to platelet aggregation and thromboembolic events in human in vitro studies,[22] suggesting another mechanism by which PD may trigger ASCVD events. *Streptococcus sanguis*, frequently isolated from dental plaque, has been noted in animal studies to activate circulating platelets during bacteremia, forming thromboemboli, causing transient myocardial ischemia via coronary occlusion [21]. Subsequent studies in humans have reported that PD is independently associated with increased blood platelets,[23] and that periodontal treatment prevents accelerated platelet activation in patients with PD [24]. Awareness of this heightened platelet response supports the current clinical practice of continuing antiplatelet agents during routine dental procedures.

### 3.5 Endothelial and other direct vascular effects

There is also indirect evidence implicating endothelial dysfunction in PD. Studies in patients with severe PD have shown attenuated flow-mediated dilatation of the brachial artery compared with matched controls [25]. Tonetti et al. observed that intensive periodontal treatment resulted in improved endothelial function (i.e., increased brachial artery flow) [26]. Periodontal bacteria, *Actinobacillus actinomycetemcomitans* and *P. gingivalis*, have been found to penetrate human vascular endothelium [27] and cause endothelial dysfunction via LPS-mediated effects [28]. PD may also contribute to the formation of reactive oxygen species (ROS) within vessel walls, and periodontal treatment thereby reduces biomarkers of oxidative stress [29]. PD may enhance the number of proteoglycans in the intimal extracellular matrix and promote entrapment of remnant lipoproteins within the intima [30]. Lastly, periodontal inflammation has been associated with imbalances in the synthesis of endogenous vasodilators (e.g., nitric oxide, prostacyclin) and with changes in arterial wall distensibility [31].

## 4 Association between periodontal disease and ascvd risk factors

Strong associations have been noted between PD and vascular risk factors, leading to the development of the *common soil* hypothesis. This concept postulates that ASCVD risk factors, including physiologic and behavioral risk factors, are common in both diseases and trigger inflammation in both gingival tissue and arterial walls, leading to PD as well as ASCVD events. It is also important to keep in mind that these relationships may be bidirectional in that these disease states could also potentially worsen specific risk factors. ASCVD risk factors linked to PD are discussed below, and raise the important question of whether efforts to mitigate these shared risk factors could also reduce the risk of both PD and ASCVD.

### 4.1 Diabetes mellitus

There is strong evidence to support a bidirectional relationship between PD and diabetes mellitus (DM). In the PAROKRANK study, undetected dysglycemia was independently associated with both MI and severe PD, and doubled the risk of both [32]. Chronic inflammation, which is observed with PD, is also associated with insulin resistance and worsening glycemic control [33]. Aggressive treatment of PD is associated with improved glycemic control in patients with type 2 DM and improved vascular function as measured by brachial artery flow-mediated dilatation [34,35]. A single-center, parallel-group, randomized control trial by D'Aiuto et al. that randomized 264 patients with type 2 DM and moderate-to-severe periodontitis to intensive periodontal treatment (IPD) versus conventional periodontal treatment (CPT) and showed that IPD was associated with improvements in HgA1c levels at 12-months compared with community-based oral care [34].

### 4.2 Cigarette smoking and vaping

Cigarette smoking and electronic cigarettes are both associated with significant increases in the risk of CVD events. Both are also associated with PD, oral bone loss, gingivitis, and tooth loss [36]. Presumed mechanisms are increases in oxidative stress and inflammatory cytokines [37]. In large retrospective studies, smoking 15 or more cigarettes per day was associated with an OR of 3.64 (95% CI, 3.00 - 4.42) for tooth loss in men and 2.47 (95% CI, 2.11 - 2.89) in women after adjustment for multiple potential confounders [38]. Similarly, based on data from 12,325 US adults in the NHANES, a strong risk of PD was observed among young smokers (adjusted OR 18.55; 95% CI 9.44 - 36.45) compared to non-smokers [39]. For smokers 50 years or older, the PD risk was even higher (adjusted OR 25.64; 13.04 - 50.40). Oral microflora, and the inflammatory response to dental plaque biofilm, appear to be affected by smoking.^36, 38^\

### 4.3 Dyslipidemia

PD is associated with pro-atherogenic changes in lipoprotein metabolism, as reflected in increased plasma levels of non-esterified fatty acids, cholesterol, and triglycerides (TGs) [15]. A 2017 meta-analysis noted a significant positive association between PD and elevated blood levels of low-density lipoprotein cholesterol (LDL-C) and TGs, and lower levels of high-density lipoprotein cholesterol (HDL-C), after adjusting for potential confounders, including diabetes and smoking [40]. LDL particles in patients with PD are reported to be smaller, denser, and more atherogenic than patients without PD [41]. Nishimura et al. notes human inflammatory cytokines released in response to *P. gingivalis* increase the expression of 3‑hydroxy-3-methylglutaryl coenzyme A (HMG-CoA) reductase, thereby increasing hepatic cholesterol synthesis and circulating levels of very low-density lipoprotein (VLDL) and LDL [42]. In observational studies, statin treatment has been associated with a reduced incidence of PD [43]. These findings may relate to the anti-inflammatory effects of statins; however, the potential for confounding due to better access to health care cannot be excluded [43]. It should also be noted that periodontal therapy has not been found to have an impact on lipid parameters [44].

### 4.4 Hypertension

Data from the National Health and Nutrition Examination Survey (NHANES) from 2009 to 2014 supports a positive association between elevated blood pressure (BP) and severe PD [45]. A recent systematic review of 40 publications, including three cohort studies, showed that the odds of having hypertension were significantly higher in patients with periodontitis than without periodontitis [46]. Furthermore, there is a positive linear association between increasing severity of periodontitis and hypertension. A 2018 study of 3600 participants with hypertension found that those with PD were less likely to respond to anti-hypertensive medications and 20% less likely to achieve BP control [45]. In the Korea National Health and Nutrition Examination Survey (KNHANES), data from approximately 20,000 participants from Korea between 2008 and 2010 were analyzed to determine the relationship of the prevalence and control rate of hypertension and numerous variables, including oral hygiene behavior assessed by questionnaires. Using a multivariate analysis and adjusting for various factors, they found that systolic BP was lower in those who reported an increase in tooth brushing frequency and the use of secondary oral hygiene products. The adjusted odds ratio (OR) of hypertension prevalence was 1.195 (95% CI, 1.033 - 1.383) for subjects who brushed their teeth hardly ever or once daily versus those who brushed after every meal. They concluded that poor oral hygiene may be an independent risk factor for hypertension and implementing good oral hygiene behaviours may help prevent and control hypertension [47]. A biologically plausible mechanism may be an improvement in inflammation and, therefore, endothelial function.

### 4.5 Obesity

Obesity is associated with chronic inflammatory diseases such as arthritis, diabetes and cardiovascular disease and similarly, is associated with higher rates of PD. In one study, obesity was associated with two times the rate of PD than people with a normal BMI [48]. An early study looking at NHANES III data (*n* = 13,664) found a correlation between BMI and PD in younger adults, but not middle-age or olders adults [49]. The OR of having PD for BMI <18.5 kg/m^2^, 25−29.9 kg/m^2^, and ≥30 kg/m^2^ were 0.21, 1.00 and 1.76 respectively. Younger patients with high waist circumference had an OR of 2.27 for having PD. Overall obesity and abdominal obesity were linked to an increased prevalence of PD, especially in patients aged 18 to 34, and may represent a critical population that would benefit from the promotion of healthy nutrition and adequate physical activity.

The links between obesity and PD may lie in common risk factors including specific dietary components, associated diabetes or suboptimal lifestyle behaviors overall. However, it is also feasible that the systemic inflammation associated with obesity contributes to PD [44].  Adipocytes contain activated macrophages and as adipocytes become more abundant and larger, these activated macrophages trigger production of cytokines, such as interleukins and tumor necrosis factors [50,51]. It is these inflammatory cytokines which may increase a host's risk for periodontal breakdown.

### 4.6 Dietary quality

Dietary patterns are associated with both oral and cardiovascular health, with similar diets linked to benefits in both conditions. Compared to a Western dietary pattern, diets high in fruit and vegetable intake have been associated with a lower risk of PD [52]. Fiber intake is associated with lower prevalence and severity of PD [53]. Whole grain intake is also inversely associated with incident PD [54]. This relationship was postulated to be, in part, due to the fiber content of whole grains leading to decreased systemic inflammation [54]. In contrast, refined carbohydrates are positively associated with dental caries, and PD [55]. Saturated fats are positively associated with PD in a dose-dependent manner, possibly mediated through increased inflammation [56].

## 5 Association between periodontal disease and ASCVD

Studies in humans have observed a positive association between PD and ASCVD since the late 1980s [57]. PD has been associated with an increased overall ASVCD risk of an estimated 3.5 fold,[58] and a 24–35% increased risk of acute CHD events [59]. In a meta-analysis of 22 studies that included 129,630 participants, PD increased the risk of MI by 2-fold (pooled OR 2.02, 95% CI, 1.59–2.57) after adjusting for cardiovascular risk factors [60]. Likewise, a meta-analysis of 7 studies that individually adjusted for a variety of risk factors, found that PD was a significant risk factor for developing peripheral arterial disease [61]. A positive association between PD and incident ischemic stroke was observed among 10,362 adults after controlling for age, cardiovascular risk factors, and socioeconomic characteristics [62]. PD severity has been observed to be associated with an increased risk for ischemic stroke [62].

Although the association between PD and ASCVD was noted to hold true after adjustment for demographic and cardiovascular risk factors in the above studies, several studies have noted attenuation in the association between PD and ASCVD after adjustment for demographic and cardiovascular risk factors [63]. Park et al. observed an increased risk of ASCVD events (a composite of CV mortality, non-fatal MI, HF, and non-fatal stroke) among 247,696 Korean adults with PD compared to those without PD over 10-years of follow-up [64]. Although this association was no longer statistically significant after adjustment for age and cardiovascular risk factors, the number of missing teeth, dental caries, daily tooth brushings, and professional cleanings all remained significantly associated with cardiovascular outcomes [64].

## 6 Association between periodontal disease and subclinical ASCVD

In an effort to understand the increased ASCVD rates noted, it is also important to understand whether PD is associated with subclinical disease. A recent large prospective cohort study showed that, during a median follow-up of 27 years, there was a positive link between childhood oral infections and subsequent subclinical atherosclerosis [65]. The presence of periodontitis in a cohort of 755 children was associated with a RR of 1.69 (95% CI 1.21 - 2.36) for the development of increased carotid artery intima-media thickness (IMT) in adulthood. In addition, a recent study of 304 patients who underwent PET imaging for cancer screening and had no clinical CVD at the time of the PET scan showed that PD was associated with a 2.25-fold increased risk (95% CI 1.47 - 3.44, *p* < 0.001) of developing a major adverse cardiovascular event, which was believed to be secondary to arterial inflammation in 80% of cases [66]. Although these observations suggest a causal mechanism, it has yet to be confirmed.

## 7 Treatment of pd to reduce cvd risk

Periodontal therapy may include dental hygiene, surgical approaches (e.g., deep scaling and root planning), and in some cases, antibiotic management. In a study in which dental hygiene was held for 21 days in young adults, improvements in markers of systemic inflammation (hsCRP, IL-6, and MCP-1) were observed when dental hygiene was resumed [67]. A recent review also noted that nonsurgical periodontal treatment may reduce levels of systemic inflammatory markers in the short-term, potentially influencing atherogenesis and resolving inflammatory oral foci [68].

Despite the observational data that links PD to the risk of ASCVD, evidence that treatment of PD reduces hard cardiovascular events is limited. A recent Cochrane review noted a single RCT of patients with ASCVD and PD that examined whether surgical intervention for PD (i.e., deep scaling and root planning) reduced inflammation and ASCVD events compared to maintenance treatment (oral hygiene instruction with recommendations to see a dentist) [69]. The use of low-dose antibiotics such as doxycycline can decrease PD by inhibiting MMPs, suggesting a mechanism that could reduce ASCVD events [70]. However, data examining the use of doxycycline for the prevention of ASCVD progression is not conclusive and routine use of antibiotics without a clear indication should be avoided due to the risks of side effects, drug-resistance and changes in the microbiome [70]. Other agents targeting inflammation such as aspirin, statins, and omega-3 fatty acids in treating PD show promise;[43,71] however, additional data is warranted.

## 8 A call to action: leveraging public awareness, literacy and health care reform to improve the risk of pd and future CVD

The observed relationship between PD and ASCVD has important public health implications. Both are highly prevalent and disproportionately affect those with lower socioeconomic status (SES). In a report by the Institute of Medicine in 2011, they recommended that interprofessional, team-based dental care has the potential to improve care-coordination, patient oucomes and produce cost savings [72].

The noted link between poor oral health and SES appears to be due to both low patient awareness and greater barriers to regular dental care, with low to middle income families affected the most [73]. A systematic review of 41 predominantly cross-sectional studies found that the number of dental caries was associated with education, income, and occupation [74] as well as low health literacy [75,76], yet data also suggest these disparities are not reduced when barriers to oral care are eliminated [76]. Therefore, patient education by clinicians and the public health sector are likely needed to raise awareness of the importance of dental health for reducing future CV disease (Table 1). Infographics and tailored print information via patient portals from dental and cardiology professionals (e.g., like the American College of Cardiology CardioSmart portal), as well as digital education and messaging, and mobile device and applictaions, could play important roles.

**Table 1:** A call to action.

Action Item	Description	Potential Impact
Clinician Education	Increase clinician awareness of the association between PD and CVD to facilitate screening practices, education of patients, and early referrals.	Early implementation of preventative care strategies to reduce PD and CVD risk.
Screening for CVD in Dental Practices	Recognition of CV Risk factors in PD patients (including hypertension, obesity and smoking screening) accomplished with BP checks and weights with BMI calculation in the dental office.	Increase preventive and treatment strategies for undiagnosed CVD
Screening for PD in Medical Offices (including cardiology)	Recognition of PD risk factors in CVD patients with reminders to visit twice annually for dental checkups, periodic questioning about oral health with a focus on oral health during the physical exam.	Increase the preventive and treatment strategies for undiagnosed PD
Improved Communication/ Collaboration	Enhance communication between dentists, physicians, and patients in a multidisciplinary model by obtaining the name of the dental and physician providers at check in, sharing office / progress notes and holding interdisciplinary conferences.	Improved quality of care and opportunity for collaborative health care solutions as well as research investigation.
Shared Data/EMR	Use of shared EMR to facilitate information exchange. The future may allow for increased interoperability between dental and medical EMRs.	Increase the recognition of risk factors that might otherwise be missed with more automated alerts.
Lifestyle Programs	Increased availability of accessible programs for lifestyle modification (smoking cessation, healthier eating, weight loss, blood pressure management)	Improved oral and CV health attainment.
Health Care Reform	Advocacy by clinicians, medical and dental organizations for increased patient coverage through Medicaid and Medicare plans.	Expand coverage for preventive care and treatment of PD to lessen the downstream associated disease

Improving clinical awareness of patient populations at risk for PD-associated CV events is also needed, and could lead to better screening, prevention and treatment. As noted, oral health has not been a major focus of clinical cardiologists or CV team members, except for patients planning valve surgery [73]. Routine screening, identification and treatment of PD in post-MI patients, triggered by cardiology referrals to dentists, could potentially reduce the risk of recurrent ischemic events. Better education of dental clinicians about the link between PD and CVD could provide opportunities for screening of CVD risk factors among underserved dental patients with reduced access to other medical care, and could potentially improve health and economic outcomes. Such educational efforts should start within medical and dental school curricula. Recent modeling data have shown that dental practice-based screening for high BP, cholesterol and glucose in adults age 40 and older who have not seen a physician in more than 12 months could save the health care system between $42.4 million ($13.51 saved per person screened) and $102.6 million ($32.72 saved per person screened) over one year [77].

Better communication and integration between medical and dental specialists could also improve CVD outcomes linked to PD. Medical and dental care are currently fragmented and in need of better integration nationally and locally, with shared Electronic Health Records (EHR's) potentially playing a larger role. Lastly, interdisciplinary conferences are needed to determine the best strategies for clinical collaborations and to identify needs for future research on the association between oral health and ASCVD.

Better health policies that impact dental health are also needed. In the US, few dental providers participate in Medicaid; traditional Medicare Part A and B plans provide no dental coverage except for that performed in a hospital; and dental care “deserts” exist in many rural areas [78]. Broader use of Medicare Advantage (Part C) plans, which now cover most preventive dental services, and increased Medicaid coverage for low income individuals (currently only in 17 states) could reduce disparities in dental care among the 50 million elderly Medicare beneficiaries in the U.S, underserved populations and those purchasing individual health insurance plans [78].

## 9 Future research

The current review is limited by the inability to establish causality between PD and CVD in prior studies due to confounding factors from multiple shared risk factors and pathophysiologic mechanisms. The existing data is predominantly observational, with limited sample size and very little randomized controlled trial data. In addition, the criteria and evaluation methods for PD appear to differ among studies potentially impacting outcomes.

Studies that investigate genetic susceptibility and less studied molecular mechanisms are needed to improve our understanding of the link between PD and ASCVD. It would be interesting to conduct a prospective cohort study following patients with a new diagnosis of PD and screen them for the development of clinical and subclinical ASCVD, and conversely, screen those with clinical ASCVD for the development of PD. There is also a need for high quality data from randomized controlled trials to evaluate whether PD contributes to the development of ASCVD after controlling for confounding factors.

## 10 Conclusions

Despite the lack of causality, PD and ASCVD are inflammatory diseases that share many common pathophysiological mechanisms and risk factors. However, efforts aimed at improving awareness and prevention of PD as a risk factor for ASCVD should not be delayed while awaiting greater proof of. Increased public and provider awareness, and interdisciplinary research and clinical collaborations (related to screening, prevention and referrals), combined with ongoing dental health care policy reforms may greatly reduce the high prevalence of PD and the associated burden of CVD in the U.S. population.

## Statement of authorship

Eugenia Gianos MD, Elizabeth A. Jackson MD MPH, Andrew M. Freeman MD, Kenneth E. Fleisher DDS^,^ Astha Tejpal MD, Karen Aspry MD MS worked together on manuscript design, writing and editing. James O'Keefe MD, Monica Aggarwal MD, Ankur Jain MD, Dipti Itchhaporia MD,^6^Kim Williams MD, Travis Batts MD, Clark Yarber MD, Robert Ostfeld MD MS [11], Michael Miller MD [12], Koushik Reddy MD [13], each wrote sections of the manuscript. Kathleen E. Allen MS RD- assisted with writing and managing the references for the manuscript.

## Declaration of Competing Interest

EAJ Research funding: (NIH [R01AG045136, UL1TR001417, UOA-212,513, P30CA013148-47S6], Amgen; Consulting: ACC, McKesson; Roylaties: UpToDate; Editorial Board: American Heart Association; JOK major ownership interest: CardioTabs; DI Research Funding Amgen; RO Consulting: Better Therapeutics; AMF: Speaker Board Boehringer-Ingleheim; All other authors have nothing to disclose.
